# Intentional Overdose on Liquid Clonazolam Reversed with Flumazenil: A Case Report

**DOI:** 10.5811/cpcem.59853

**Published:** 2023-11-08

**Authors:** Gayle Galletta

**Affiliations:** UMass Chan Medical School, Department of Emergency Medicine, Worcester, Massachusetts

**Keywords:** case report, clonazolam, designer benzodiazepine, flumazenil

## Abstract

**Introduction:**

Clonazolam is a designer benzodiazepine that can be purchased illicitly on the internet. The use of designer benzodiazepines is increasing in both the United States and abroad, and patients may present to the emergency department (ED) after intentional or non-intentional overdose.

**Case report:**

This case report describes a patient who presented to a community ED after an intentional overdose on liquid clonazolam and was successfully treated with flumazenil.

**Conclusion:**

Since the pharmacologic action of clonazolam is similar to benzodiazepines, the sedative-hypnotic effect can be reversed with flumazenil, a benzodiazepine antagonist.

CPC-EM Capsule What do we already know about this clinical entity?

*Clonazolam is a designer benzodiazepine that was first synthesized in 1971. Its toxicity was first identified in 2016.*

What makes this presentation of disease reportable?

*To date there have been no reports of clonazolam toxicity in the emergency medicine literature nor of its reversal with flumazenil.*

What is the major learning point?

*Designer benzodiazepines use is increasing, and patients may present to the ED after an overdose. The sedative-hypnotic effect can be reversed with flumazenil.*

How might this improve emergency medicine practice?

*This case raises the awareness of designer benzodiazepine, its easy availability online, and the ability to reverse its adverse effects with flumazenil.*

## INTRODUCTION

Designer drugs, also known as novel psychoactive substances, are synthetic analogs of a controlled substance that are designed to mimic the effect of the original substance while avoiding regulation and law enforcement.^1^ Clonazolam is a designer benzodiazepine that was first synthesized in 1971.^2^ Clonazolam toxicity was identified in Europe in 2016 and in the United States in 2017.^3^ The psychiatry community sounded the alarm in 2015,^4^ and the toxicology community has been reporting and following the trends of designer benzodiazepines toxicity.^1^ ^,^ ^3^ ^,^ ^4^ ^–^ ^6^ However, there have been no reports to date of clonazolam toxicity in the emergency medicine literature.

This case report describes a patient who took an intentional overdose of concentrated, liquid clonazolam. He presented to a community ED with a sedative-hypnotic toxidrome and was managed successfully with flumazenil.

## CASE REPORT

**Image 1.**
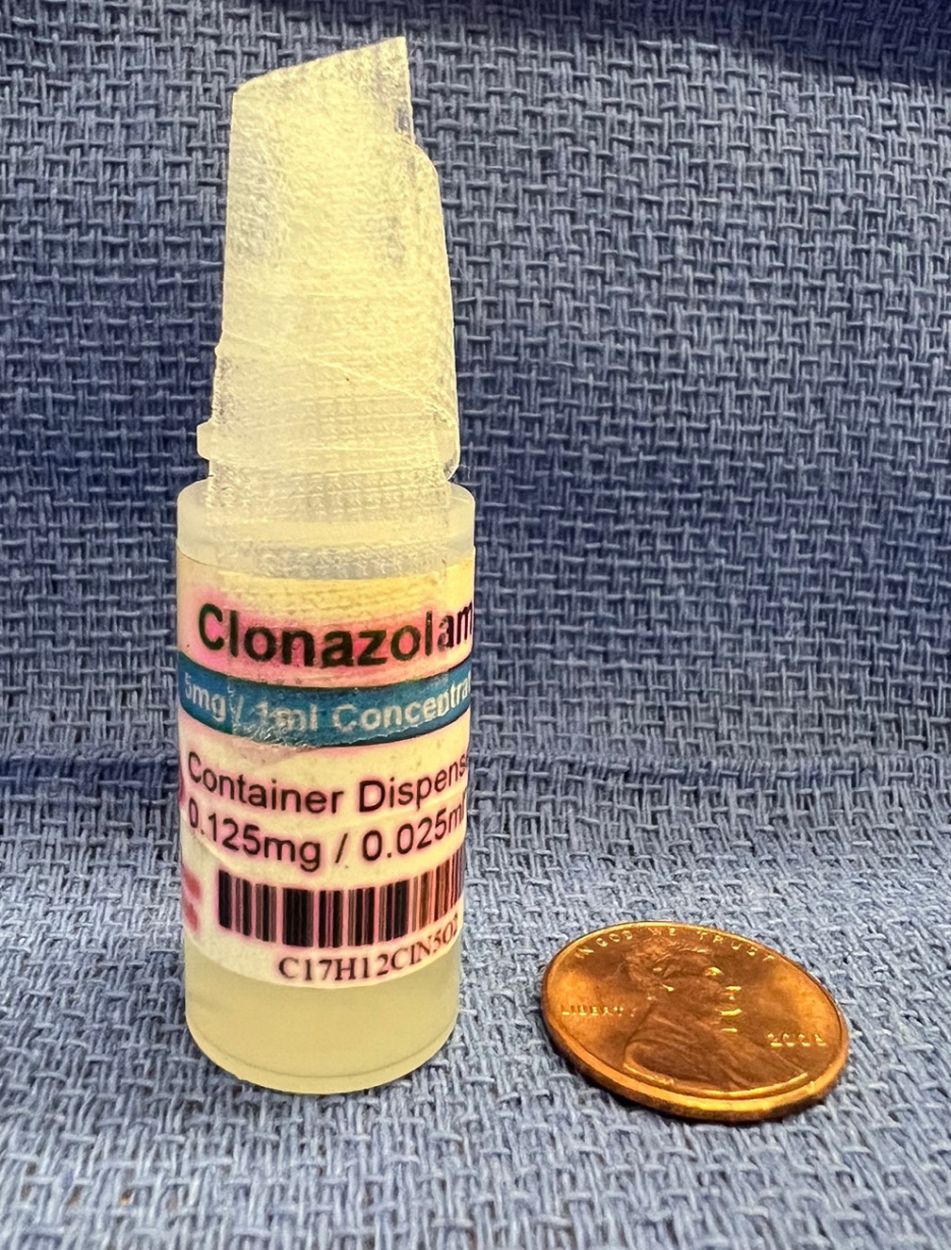
Bottle of clonazolam ingested by patient.

A 31-year-old male with a history of untreated depression and alcohol abuse was brought by ambulance to the emergency department (ED) for somnolence after an intentional overdose of liquid clonazolam, which he had received from a friend. The patient had sent a suicidal text to his ex-girlfriend shortly before he ingested approximately half of a three-milliliter bottle of liquid clonazolam (Image 1). When she arrived at his house, within approximately 30 minutes of his text, he appeared confused and intoxicated, and she called emergency medical services (EMS). Upon EMS arrival, the patient was somnolent. His fingerstick blood sugar was 108 milligrams per deciliter (mg/dL) (reference range 70–100 mg/dL). When the patient arrived at the community ED, his blood pressure was 115/79 millimeters of mercury, heart rate 89 beats per minute, respiratory rate 22 breaths per minute, and oxygen saturation of 94% on room air. He was somnolent and minimally following commands but was protecting his airway. Pupils were 4 millimeters, equal, round, and reactive to light. He was not diaphoretic. Lungs were clear, and heart was regular rate and rhythm. The abdomen was soft and nontender, and bowel sounds were present. He had normal patellar reflexes without clonus. There were a few old, superficial abrasions to the left forearm. Glascow Coma Score was 12 (minus three for verbal response).

Lab work was significant for a low normal bicarbonate of 22 milliequivalents per liter (mEq/L) (reference range 22–29 mEq/L). The anion gap was normal. Potassium was slightly low at 3.2 millimoles per liter (mmol/L) (3.6–5.2 mmol/L). Salicylate and acetaminophen levels were undetectable. Ethanol was 204 mg/dL. Urine drug screen (UDS) was positive for cocaine. It should be noted that benzodiazepines are not evaluated on the hospital’s UDS. An electrocardiogram (EKG) was non-ischemic with normal intervals.

Approximately one hour after arrival in the ED (90 minutes after ingestion), the patient became more somnolent with respiratory depression and oxygen saturations in the mid-80s. Flumazenil 0.2 mg intravenous (IV) was administered with immediate improvement in respiratory effort and oxygenation. One hour later, an additional 0.2 mg dose of IV flumazenil was again administered for respiratory depression and hypoxia. Because there were no intensive care unit beds available, the patient continued to board and be managed in the ED. Within six hours, his mental status normalized, and he was medically cleared for psychiatric evaluation. The next day, the patient recounted taking the clonazolam as a suicide attempt. He recalled drinking alcohol and perhaps using cocaine.

## DISCUSSION

Clonazolam (6-(2-chlorophenyl)-1-methyl-8-nitro 4H-triazolo[4,3-α] benzodiazepine) is an analog of clonazepam.^2^ It can be found in tablet, capsule, pellet, blotter, and liquid form, and can be purchased on the internet.^5^ Clonazolam is considered a designer benzodiazepine (along with dozens of others)^1^ that has no medicinal indication and is not currently regulated by the US Food and Drug Administration (FDA). In December 2022, the FDA published a temporary order to add five synthetic benzodiazepines (clonazolam, etizolam, flualprazolam, flubromazolam, and diclazepam) to Schedule 1 under the Controlled Substances Act.^7^

Since clonazolam behaves similarly to benzodiazepines, it is likely safe to assume that it could be reversed by flumazenil, a benzodiazepine antagonist which “competitively inhibits the activity of benzodiazepine and non-benzodiazepine substances that interact with benzodiazepine receptors site on the gamma-aminobutyric acid (GABA)/benzodiazepine receptor complex. It can also reverse the binding of benzodiazepines to benzodiazepine receptors.”^8^ Typical onset of action is 1–2 minutes with an 80% response rate within three minutes. Its peak effect is 6–10 minutes with a duration of 19–50 minutes.^8^ There is currently a black box warning for flumazenil in the US as there has been a correlation with seizures, especially in patients on benzodiazepines long term, and in those with severe tricyclic antidepressant overdose.^9^ Flumazenil is used more liberally in Europe. The package insert from Europe states not to use flumazenil if it is being administered to control a potentially life-threatening situation such as elevated intracranial pressure or a serious epileptic seizure. It also warns not to use flumazenil in mixed intoxications involving tri- or tetracyclic antidepressants, as the toxicity of the antidepressants can be masked by the protective benzodiazepine effects.^10^

The patient presented here had a co-ingestion of highly concentrated clonazolam (approximately 7.5 mg) along with ethanol and cocaine. There is no regulated dose for clonazolam, but profound sedation is thought to occur at doses of 0.5 mg.^11^ The cocaine may have counteracted some of the sedative effects of the clonazolam; however, the patient could not later recall the precise timing of his cocaine use. Since he had no known long-term use of benzodiazepines, the decision to try reversal with flumazenil was chosen as opposed to intubation. The treating emergency physician is licensed in both the US and Europe and, therefore, was comfortable and experienced with its use. This patient responded favorably to the flumazenil within the expected time frame and had no resultant seizure activity.

## CONCLUSION

The use of designer benzodiazepines is increasing, and patients may present to the ED after accidental or intentional overdose. The sedative-hypnotic toxidrome is similar to benzodiazepine overdose and, after assessment of risk vs benefit, flumazenil may be helpful in its reversal.
